# A Meta-Analysis of Arsenic Trioxide Combined with Transcatheter Arterial Chemoembolization for Treatment of Primary Hepatic Carcinoma

**DOI:** 10.1155/2016/3428370

**Published:** 2016-06-13

**Authors:** Ling He, Qingyun Xu, Liguo Chen, Ruixue Chen

**Affiliations:** Department of Chinese Medicine, Medical College of Jinan University, Guangzhou, Guangdong 510632, China

## Abstract

Primary hepatic carcinoma (PHC) is one of the most common malignant tumours in the world. More and more research has shown that As_2_O_3_ combined with TACE has a good curative effect in treating PHC. The objectives of this study were to evaluate the therapeutic efficacy and safety of As_2_O_3_ combined with TACE in treating PHC. The CNKI, VIP, Wanfang, PubMed, and Cochrane databases were searched from their inception until December 2015. Randomized controlled trials (RCTs) comparing As_2_O_3_ combined with TACE versus TACE alone in treating PHC were identified. Stata SE 12.0 was used for data analysis. 17 RCTs with 1055 patients were included. Meta-analysis showed that, compared with TACE alone, As_2_O_3_ combined with TACE showed significant effects in improving the clinical efficacy rate (*P* < 0.01), decreasing the value of alpha-fetoprotein (*P* < 0.01), increasing the one-year survival rate (*P* < 0.01), and improving the quality of life of PHC patients (*P* < 0.01). Fifteen studies had mentioned adverse events, but no serious adverse effects were reported in any of the included trials. In conclusion, As_2_O_3_ combined with TACE therapy appears to be potentially effective in treating PHC and is generally safe. However, further studies with rigorous designs trials and multiregional cooperation trials are needed.

## 1. Introduction

Primary hepatic carcinoma (PHC) includes hepatocellular carcinoma (HCC) and intrahepatic bile duct carcinoma. It is a common malignancy, and its incidence and mortality rate are increasing annually, making it a serious threat to human health.

Surgical resection is the primary therapeutic approach for PHC but is limited by the hidden onset of PHC. Because of this hidden onset, lack of specificity of the early symptoms, and rapid progress, PHC is usually diagnosed in the middle-to-late stages, when it is past the window of operability [1]. Transcatheter hepatic arterial chemoembolization (TACE) is the first-line treatment for the patients with unresectable PHC. The survival benefit of TACE is supported by the results of a meta-analysis of clinical trials comparing TACE with other conservative treatments in patients with inoperable PHC [2]. The results showed that the median survival of patients improved following TACE [3, 4]. Chemotherapy regimens commonly used in clinical settings include 5-fluorouracil, antibiotics (mitomycin, adriamycin, and pirarubicin), and platinum drugs (cisplatin and oxaliplatin). Patients are provided with symptomatic treatment to protect the liver and acid. When necessary, patients are treated to elevate albumin and white blood cells [4]. With the wide application of TACE, the drug regimen for primary hepatic embolization and chemotherapy requires diversification, and the use of traditional Chinese medicine (TCM) is increasing.

As_2_O_3_, an arsenic compound, is approved and listed as an antitumour drug in some countries. In the 1970s, Professor Zhang TD found that As_2_O_3_, the active ingredient of which is arsenic, was effective in the treatment of leukemia, and it received attention and recognition from the international medical community. A large number of animal experiments and clinical application studies in As_2_O_3_ for the treatment of liver cancer have been carried out by scholars. The research showed that As_2_O_3_ with TACE prevents recurrence and metastasis in treated PHC, primarily by changing the composition of the cancer cell nuclear matrix protein, inhibiting the expression of cancer cell proliferation cell antigens, inducing cancer cell apoptosis, and inhibiting HCC development [5]. As_2_O_3_ with TACE is used to treat PHC, as seen in the increasing number of clinical research reports. However, no meta-analyses, reviews, or systematic reviews have evaluated the benefits of As_2_O_3_ combined with TACE in the treatment of PHC. Therefore, we evaluated the therapeutic efficacy and safety of As_2_O_3_ combined with TACE in the treatment of PHC on the basis of existing clinical studies.

## 2. Materials and Methods

### 2.1. Search Strategy

To ensure a complete search, we carried out a comprehensive investigation of available periodical databases and limited the search from the day of inception to December 2015. We used “arsenic trioxide (or) As_2_O_3_,” “liver cancer (or) liver neoplasms,” “primary hepatic carcinoma,” and “transcatheter arterial chemoembolization (or) TACE” as keywords to retrieve studies from PubMed, Cochrane, the Chinese science and technology periodical full-text database of CNKI, the Chinese medical information resources system of VIP, and the Wanfang database.

### 2.2. Inclusion Criteria

#### 2.2.1. Types of Studies

 A randomized controlled trial (RCT) of As_2_O_3_ combined with TACE for the treatment of PHC, including a comprehensive statistical index and complete general information, is included.

#### 2.2.2. Object of Studies

The objects of the studies were in accordance with the standard of defined PHC. The diagnosis criteria of PHC were based on the new methods for the diagnosis and treatment of common malignant neoplasms [5]. All cases were confirmed by pathology, cytology, and/or an imaging examination diagnosis of patients with advanced PHC.

#### 2.2.3. Interventions

The control groups were treated with TACE. The experimental groups were treated with As_2_O_3_ combined with TACE, excluding those treatments that were combined with other medication studies.

#### 2.2.4. Index of Observation

The main outcome indicators included the following: (a) the therapeutic responses, categorized according to WHO criteria as follows: complete response (CR), partial response (PR), stable disease (SD), and progressive disease (the objective efficient = (the cases of complete response + the partially catabatic cases)/the total number of cases multiplied by 100%); (b) alpha-fetoprotein (AFP): the AFP drop ratio = the cases of AFP decreasing after treatment/the cases of AFP increasing before treatment; (c) survival period: 1-year survival rate = the cases of survival/total in the first year; (d) the quality of life: rate of healing increase = the number of cases with a Karnofsky score of 10 points or more after treatment/total number multiplied by 100%; (e) adverse reactions.

### 2.3. Standard of Exclusion

Studies that met any of the following criteria were excluded: (a) the inclusion of cases of metastatic HCC; (b) non-RCTs studies; (c) studies that used As_2_O_3_ combined with other positive drugs; and (d) control groups that were given TACE and other treatments.

### 2.4. Screening of Included Texts

Two reviewers independently read the titles and abstracts of the potential studies. They excluded the studies that obviously did not meet the inclusion criteria. They read the full texts of studies that met the standards to determine whether they truly met the inclusion criteria. They then cross-checked the texts and discussed disagreements or suggestions for solutions.

### 2.5. Quality Evaluation

We used the Cochrane Handbook 5.0.1 bias risk assessment tools to evaluate the quality of each included study [6]. The assessment criteria primarily included (a) random sequence generation (selection bias), (b) allocation concealment (selection bias), (c) blinding of participants and personnel (performance bias), (d) blinding of outcome assessment (detection bias), (e) incomplete outcome data, (f) selective reporting (reporting bias), and (g) other biases.

### 2.6. Extraction

Two researchers developed a form based on the inclusion and exclusion criteria to extract the following: (a) general information, including author, date of publication, age, gender, procedures, index of observation, and adverse reactions, and (b) the type of literature quality evaluation, including author, date of publication, random method, hidden allocation scheme, blind method, description of last visit, and baseline.

### 2.7. Statistical Processing

We used Stata SE 12.0 analysis software for the statistical analyses of the selected studies. The relative risk (odds ratio (OR)) was used to analyse the statistical data, and the weighted mean difference was used to analyse the results. The effects were expressed using 95% confidence intervals (CIs). First, we tested for heterogeneity of the included studies using the chi-square test. When studies in the group were not heterogeneous (*P* ≥ 0.1, *I* ^2^ ≤ 50%), we used the fixed-effects model for the meta-analysis. When heterogeneity (*P* < 0.1, *I* ^2^ > 50%) was present, we analysed the reason and used sensitivity analyses to process the data before excluding studies of lower quality and evaluating the stability of the results of the meta-analysis. The studies whose heterogeneity still could not be eliminated were consolidated with a random-effects model. We used a forest map to list the results and an intention-to-treat analysis to determine whether there was attrition bias. However, if there was clear clinical heterogeneity among the studies, we did not merge them but rather performed a descriptive qualitative analysis. The funnel plot test was used to examine the existence of publication bias.

## 3. Results

### 3.1. The Results of the Literature Retrieval and Screening

**Figure 1:**
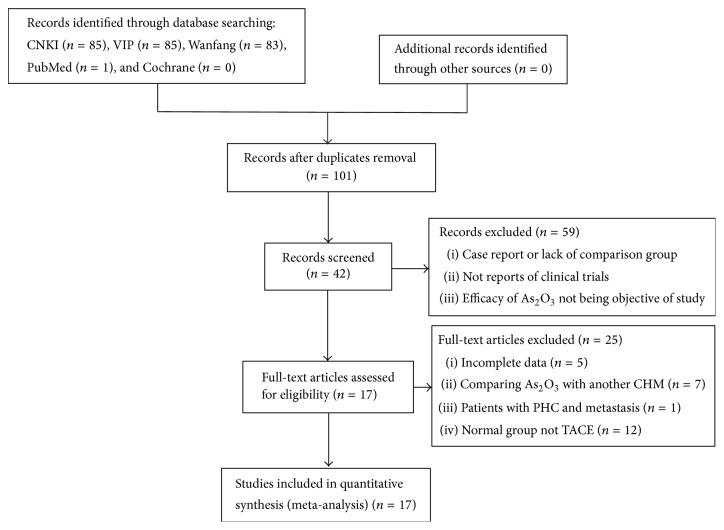
The chart of literature filtering flow.

We initially retrieved 254 articles. The CNKI database included 85 articles, the VIP database included 85 articles, the Wanfang database included 83 articles, PubMed included 1 article, and the Cochrane database included no articles. Initially, 101 articles were excluded due to duplicate publications, which were determined by reading the title, abstract, and text. In addition, we screened those studies that did not meet the inclusion criteria or used randomized contrast groups and different measurement indexes. Finally, we identified 17 articles that met the inclusion criteria. The literature filtering flow is shown in Figure 1.

### 3.2. The Basic Facts of the Included Studies

There were 17 articles that met the inclusion criteria, and 1055 patients were observed, including 530 cases in the experimental group and 525 cases in the control group (see Table 1).

### 3.3. The Quality Assessment of Studies Included in the Meta-Analysis

We used the Cochrane Handbook 5.0.1 bias risk assessment tools to evaluate the quality of the included studies and found that 17 studies were randomized. There were 4 studies [4, 7–9] that used the random number table method to divide groups and 1 [4] that used allocation concealment. Three studies [10–12] reported the situation of loss to follow-up. The research methods in the other studies were not described (see Table 2).

### 3.4. Assessment for Risk of Bias

**Figure 2:**
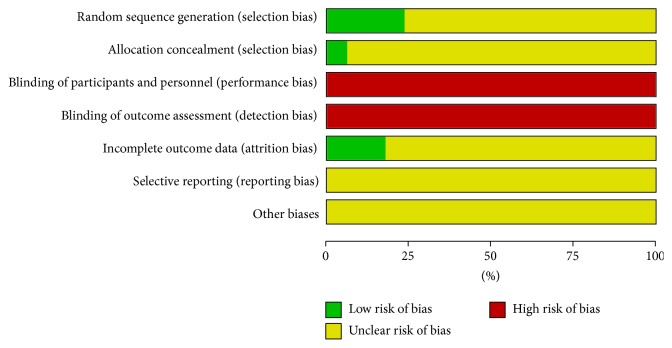
Bias risk assessment chart.

We used the Cochrane Handbook 5.0.1 bias risk assessment tools to evaluate the quality of the included studies and found that 17 studies were randomized. There were 4 studies that used the random number table method to divide the groups; and 1 study report used allocation concealment; and 3 studies reported the situation of loss to follow-up. The research methods in the other studies were not described (Figure 2).

### 3.5. Analyses of Clinical Efficacy

#### 3.5.1. The Total Efficiency of Clinical Effect

**Figure 3:**
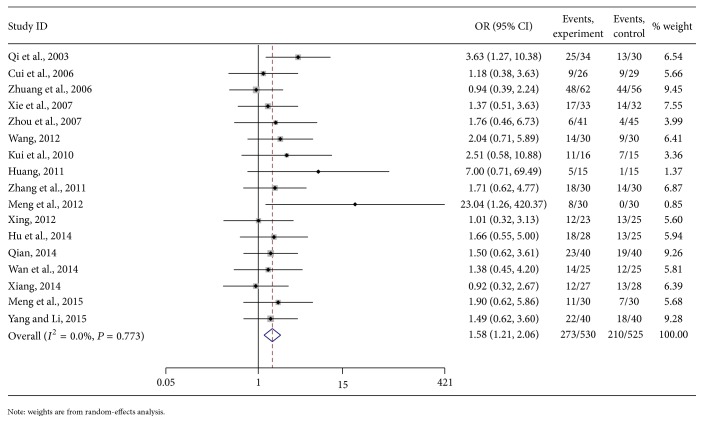
Meta-analysis on the total effects of As_2_O_3_ with TACE in treating PHC.

The meta-analysis showed that the total efficiency of the clinical effect was not heterogeneous among the included studies. According to the fixed-effects model analyses, the combined OR of the treatment and control groups was 1.58 [95% CI (1.21, 2.06), *P* < 0.01]. The diamond was on the right side of the middle line. From test *Z*, the difference between the 2 methods in PHC patients was obvious (Figure 3).

#### 3.5.2. Publication Bias of Clinical Effect

**Figure 4:**
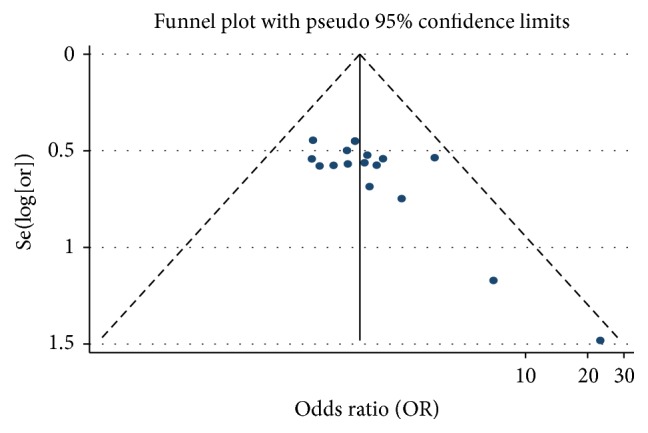
Funnel plot of the effect of As_2_O_3_ with TACE in treating PHC.

We analysed the efficacy rate of PHC treatment. We drew a funnel plot in which the curative effect ratio of the meta-analysis results in the test and control groups (OR) was used as the abscissa, while Se(log⁡[or]) was used as the ordinate. We analysed the morphology distribution of the 17 studies using a funnel plot. The distribution was not completely symmetrical around the funnel plot, which suggested the possibility of publication bias (Figure 4).

#### 3.5.3. The Effect of AFP

**Figure 5:**
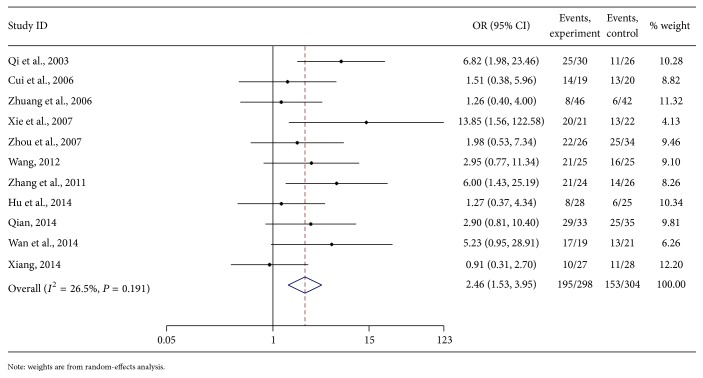
Meta-analysis on the APF of As_2_O_3_ with TACE in treating PHC.

The meta-analysis showed that the AFP of the clinical effect was not heterogeneous. According to the fixed-effects model analyses, the combined OR of the treatment and control groups was 2.46 [95% CI (1.54, 3.95)]. The diamond was on the right side of the middle line. From test *Z*, the difference between the 2 methods in PHC patients was obvious (Figure 5).

#### 3.5.4. Publication Bias of AFP

**Figure 6:**
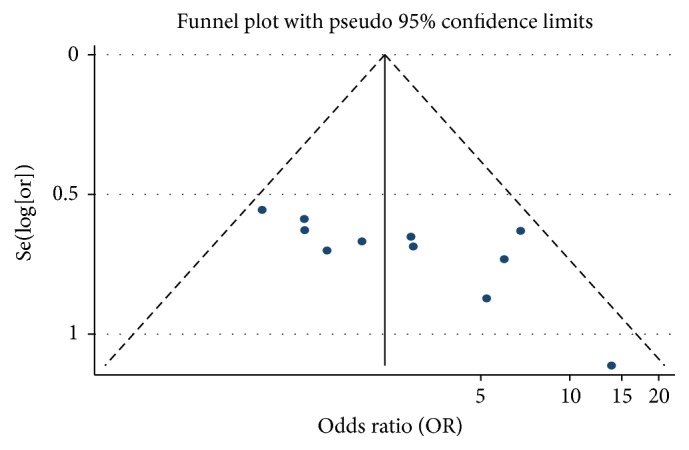
Funnel plot of APF of As_2_O_3_ with TACE in treating PHC.

We analysed the efficacy rate of PHC treatment. We drew a funnel plot in which the curative effect ratio of the meta-analysis results in the test and control groups (OR) was used as the abscissa, while Se(log⁡[or]) was used as the ordinate. We analysed the morphology distribution using a funnel plot and found it was not completely symmetrical, which suggested the possibility of publication bias (Figure 6).

#### 3.5.5. The Effect of One-Year Survival Rate

**Figure 7:**
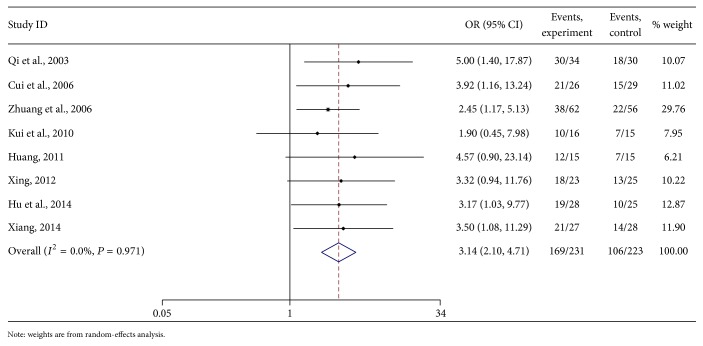
Meta-analysis on the one-year survival rate of As_2_O_3_ with TACE in treating PHC.

The meta-analysis showed that the one-year survival rate was not heterogeneous. According to the fixed-effects model analyses, the combined OR of the treatment and control groups was 3.14 [95% CI (2.10, 4.71), *P* < 0.01]. The diamond was on the right side of the middle line. From test *Z*, the difference between the 2 methods in PHC patients was obvious (Figure 7).

#### 3.5.6. Publication Bias of One-Year Survival Rate

**Figure 8:**
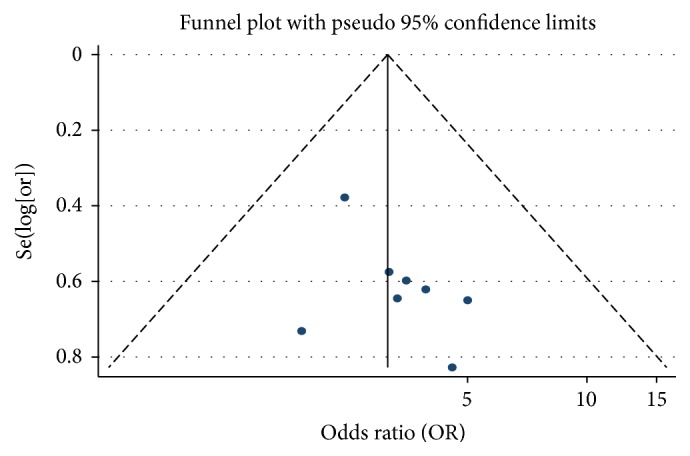
Funnel plot of one-year survival rate of As_2_O_3_ with TACE in treating PHC.

We analysed the one-year survival rate for PHC treatment. We drew a funnel plot in which the curative effect ratio of the meta-analysis results in the test and control groups (OR) was used as the abscissa, while Se(log⁡[or]) was used as the ordinate. We analysed the morphology distribution using a funnel plot and found it was not completely symmetrical around the funnel plot, which suggested the possibility of publication bias (Figure 8).

#### 3.5.7. The Effect of Life Quality

**Figure 9:**
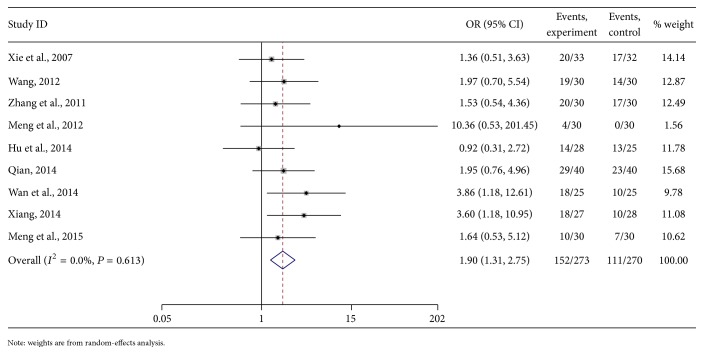
Meta-analysis on the life quality of As_2_O_3_ with TACE in treating PHC.

The meta-analysis showed that the quality of life effect was not heterogeneous. According to the fixed-effects model analyses, the combined OR of the treatment and control groups was 1.90 [95% CI (1.31, 2.75), *P* < 0.01]. The diamond was on the right side of the middle line. From test *Z*, the difference between the 2 methods in PHC patients was obvious (Figure 9).

#### 3.5.8. Publication Bias of Life Quality

**Figure 10:**
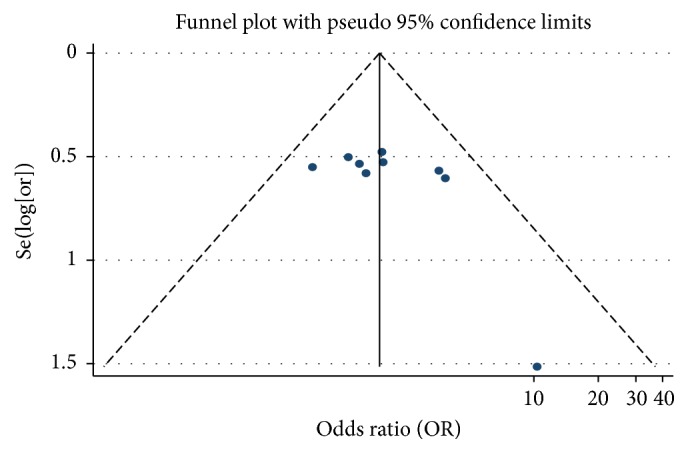
Funnel plot of the life quality of As_2_O_3_ with TACE in treating PHC.

We analysed the life quality associated with PHC treatment. We drew a funnel plot in which the curative effect ratio of the meta-analysis results in the test and control groups (OR) was used as the abscissa, while Se(log⁡[or]) was used as the ordinate. We analysed the morphology distribution using a funnel plot and found that it was not completely symmetrical around the funnel plot, which suggested the possibility of publication bias (Figure 10).

#### 3.5.9. Adverse Reactions

There were 15 studies that clearly described adverse reactions in the experimental and control groups. A variety of drug combinations may cause bloating, loss of appetite, nausea, vomiting, fever, pain, mild water sodium retention, mild oedema of the face and lower limbs, itchy skin, gastrointestinal tract reactions, haematological toxicity, and liver function damage. However, the adverse reactions were generally I~II degrees, and no degree IV reactions were noted. After timely and effective treatment, there were no serious complications, treatment was tolerated, and no treatment-related deaths occurred. Therefore, the TACE combination therapy is effective for the treatment of PHC.

## 4. Discussion

In the present study, a total of 17 articles were analysed to evaluate the effect of As_2_O_3_ with TACE in PHC patients. This meta-analysis included 1055 patients; of these, 530 patients received As_2_O_3_ combined with TACE therapy and 525 patients received TACE therapy. The results showed that As_2_O_3_ combined with TACE therapy was significantly superior to TACE alone in terms of the clinical efficacy rate (*P* < 0.01). The combined therapy decreased the AFP value (*P* < 0.01), increased the one-year survival rate (*P* < 0.01), and improved the life quality of PHC patients (*P* < 0.01). There were 15 studies that clearly described adverse reactions in the experimental and control groups, but no serious adverse effects were reported in any of the included trials. Therefore, the TACE combination therapy is effective for the treatment of PHC.

PHC is one of the most common malignant tumours in the world. It ranks fifth in incidence for malignant tumours and third in worldwide mortality. Approximately 50 million patients die from PHC each year, and its incidence is increasing. Viral hepatitis, cirrhosis, and environmental factors are thought to be causally associated with PHC. Treatment of patients with unresectable PHC is conducted with TACE [22]. TACE has advantages, including fewer adverse reactions, a tumour area with a high conventional drug concentration, an obvious curative effect, and ease of establishing collateral circulation, though it needs multiple treatments. However, the conventional repeated treatment may aggravate liver damage because it generally does not cause complete necrosis of the tumour tissue. Rather, it causes ischemia and hypoxia, which lead to increases in hypoxia-including factor and vascular endothelial growth factor expression in the tumour tissues. This treatment leads to the resistance and metastasis of the tumour, and therefore the curative effect of treatment with TACE is not ideal [17]. A rising number of PHC patients resort to Chinese medicine. The use of TCM is increasing. As_2_O_3_ is approved and listed as an antitumour drug in some countries. The inhibition of apoptosis plays an important role in the generation, development, and metastasis of malignant tumours. Apoptosis is a physiological process that is important for the preservation of homeostasis and the morphogenesis of tissues [23]. Many chemotherapeutical drugs treat malignant tumours by interfering with the pathological apoptosis regulation of tumour cells. Inducing apoptosis in tumour cells is also the operational principle of As_2_O_3_, an anticancer drug that has been used in traditional medicine for many centuries [23, 24]. More and more studies show that As_2_O_3_ combined with TACE has a good curative effect in treating PHC.

Although this meta-analysis summarizes all available eligible studies comparing the use of As_2_O_3_ combined with TACE with the use of TACE alone in treating PHC, there are still some drawbacks of this study. The evidence presented in this meta-analysis is insufficient to warrant a clinical recommendation due to the generally weak methodological quality of the included studies. The number of included RCTs was relatively small and not all studies described the method of randomization. Some significant heterogeneity may have resulted from different clinical baseline characteristics and intervention protocols among the included studies. Moreover, the values of AFP are missing which are, combined with hepatic ultrasonography, the most common markers used in clinical practice. AFP is considered to be the gold standard serum marker for the screening of patients who are at high risk of PHC, as well as for the monitoring of treatment response [25]. The weaknesses of this paper are a result of the inherent limitations in the primary studies. First, none of the included studies in this paper were formally registered with the WHO International Clinical Trials Registry Platform. Therefore, protocols were not available to confirm that the studies were free of selective reporting. Second, all studies included in this paper used an “A+B versus B” design in which patients were randomized to receive either As_2_O_3_ combined with TACE therapy or TACE alone, and there was no rigorous control for the placebo effect. All 17 studies claimed to be RCTs, but all of them failed to give adequate and convincing information on how the random allocation was generated and concealed, which is necessary to avoid selection bias. The studies also did not mention the blinding method that was used, which could lead to performance and detection biases. No intention-to-treat analyses were mentioned, and no dropouts were reported. The 17 studies were all published in Chinese because Chinese medicine injections are used only in China. This may have produced a bias of publication. Most of the studies used random groups, but neither the randomization method nor the random allocation scheme was described. Therefore, there may be biases in selectivity and implementation. No use of blinding was described for the contrasting groups, which led to a high probability of selective bias. Therefore, the results and conclusions in this study should be interpreted with caution, and it will be necessary to carry out high-quality, multicentre studies with large sample sizes that are regularly reported to provide for evidence-based medicine in the future.

In conclusion, the combination of As_2_O_3_ with TACE was better than TACE alone in treating PHC. The combination of As_2_O_3_ and TACE can lower AFP, increase the one-year survival rate, improve the life quality of PHC patients, and decrease the side effects of chemotherapy. However, a variety of drug combinations may cause bloating, loss of appetite, nausea, vomiting, fever, pain, mild water sodium retention, mild oedema of the face and lower limbs, itchy skin, gastrointestinal tract reactions, haematological toxicity, and liver function damage. However, the adverse reactions were mostly I~II degrees, and no degree IV reactions were reported. After timely and effective treatment, there were no serious complications, treatment was tolerated, and no treatment-related deaths occurred. Therefore, TACE combination therapy is effective for the treatment of PHC. Further research for application in clinical practice is needed. However, further studies with rigorous designs and large sample sizes as well as multiregional cooperation trials are needed.

## Figures and Tables

**Table 1:** The basic facts of inclusion in literature.

Included studies	Sex (man/woman) (E/C)	Age (E/C)	*n* (E/C)	Experiment group intervention	Control group intervention	Outcomes	Child-Pugh A/B/C (*n*)
Qi et al., 2003 [13]	28/6 24/6	32~68 36~72	34/30	As_2_O_3_ (20 mg/d, qd) + TACE	TACE (5-Fu 1 g + MMC 10 mg + Epi-ADM 60 mg + iodised oil 10–30 mL, iv, 35 days)	Clinical effect, AFP, survival	E: 28/4/2 C: 22/4/4

Cui et al., 2006 [7]	21/5 25/4	39~65 37~67	26/29	As_2_O_3_ (20 mg/d, qd) + TACE	TACE (MMC 6 mg/m^2^ + Epi-ADM 40 mg/m^2^ + iodised oil 10–30 mL, iv, 35 days)	Clinical effect, AFP, survival	E: NR C: NR

Zhuang et al., 2006 [10]	44/18 36/20	26~76 24~79	62/56	As_2_O_3_ (20 mg/d, qd) + TACE	TACE (cisplatin 50 mg + MMC 10 mg + Epi-ADM 50 mg + iodised oil 10–30 mL, iv, 60 days)	Clinical effect, AFP, survival	E: 28/32/2 C: 25/28/3

Xie et al., 2007 [14]	25/8 23/9	21~70 21~70	33/32	As_2_O_3_ (20 mg/d, qd) + TACE	TACE (HCP 20 mg + ADM 50 mg + cisplatin 60 mg + iodised oil 5–20 mL, iv, 42 days)	Clinical effect, AFP, survival, quality of life	E: NR C: NR

Zhou et al., 2007 [15]	35/6 40/5	21~75 23~74	41/45	As_2_O_3_ (20 mg/d, qd) + TACE	TACE (5-FU 750 mg + CAP 300 mg + THP 60 mg + iodised oil 2–5 mL, iv, 28 days)	Clinical effect, AFP	E: 35/6/0 C: 37/8/0

Wang, 2012 [5]	26/4 25/5	44~64 47~67	30/30	As_2_O_3_ (20 mg/d, qd) + TACE	TACE (CAP 300 mg + MMC 10 mg + iodised oil 5–20 mL, iv, 28 days)	Clinical effect, AFP, quality of life	E: NR C: NR

Kui et al., 2010 [11]	Unclear	Unclear	16/15	As_2_O_3_ (20 mg/d, qd) + TACE	TACE (5-Fu 750 mg/m^2^ + cisplatin 60 mg + THP 20 mg/m^2^, iv, 14 days)	Clinical effect, survival	E: NR C: NR

Huang, 2011 [4]	13/2 11/4	48~68 45~71	15/15	As_2_O_3_ (10 mg/d, qd) + TACE	TACE (THP 20–40 mg + 5-Fu 500–750 mg + iodised oil, iv, 28 days)	Clinical effect, survival	E: NR C: NR

Zhang et al., 2011 [12]	24/6 25/5	28~72 31~68	30/30	As_2_O_3_ (20 mg/d, qd) + TACE	TACE (CAP 300 mg + MMC 10 mg + iodised oil 5–20 mL, iv, 42 days)	Clinical effect, quality of life	E: NR C: NR

Meng et al., 2012 [8]	22/8 19/11	36~77 23~78	30/30	As_2_O_3_ (10 mg/d, qd) + TACE	TACE (ADM 20–30 mg + capobenic 100–300 mg + iodised oil 6–20 mL, iv, 56 days)	Clinical effect, quality of life	E: NR C: NR

Xing, 2012 [9]	18/5 23/2	44~66 46~64	23/25	As_2_O_3_ (20 mg/d, qd) + TACE	TACE (Epi-ADM 40 mg + saline 2 mL + iodised oil, iv, 28 days)	Clinical effect, survival	E: NR C: NR

Hu et al., 2014 [16]	21/7 17/8	31~80 28~70	28/25	As_2_O_3_ (10–20 mg/d, qd) + TACE	TACE (oxaliplatin 150 mg + 5-FU 1.5 g + Epi-ADM 50 mg + iodised oil 10–20 mL, iv, above 28 days)	Clinical effect, AFP, survival, quality of life	E: NR C: NR

Qian, 2014 [17]	32/8 35/5	33~81 31~68	40/40	As_2_O_3_ (20 mg/d, qd) + TACE	TACE (lobaplatin 20 mg + Epi-ADM 40 mg, iv, 28 days)	Clinical effect, AFP, quality of life	E: NR C: NR

Wan et al., 2014 [18]	16/9 18/7	44~66 42~64	25/25	As_2_O_3_ (10 mg/d, qd) + TACE	TACE (Epi-ADM 40 mg/m^2^ + 5-Fu 1000 mg/m^2^ + cis-platinum 50 mg/m^2^ + iodised oil 5–20 mL, iv, above 28 days)	Clinical effect, AFP, quality of life	E: NR C: NR

Xiang, 2014 [19]	22/5 25/3	49~71 47~69	27/28	As_2_O_3_ (15 mg/d, qd) + TACE	TACE (THP 20 mg + MMC 10 mg + iodised oil, iv, above 28 days)	Clinical effect, AFP, survival, quality of life	E: 24/3 C: 24/4

Meng et al., 2015 [20]	27/3 26/4	36~76 36~72	30/30	As_2_O_3_ (10 mg/d, qd) + TACE	TACE (oxaliplatin 100 mg + ADM 20–30 mg + iodised oil 3–15 mL, iv, 14 days)	Clinical effect, quality of life	E: NR C: NR

Yang and Li, 2015 [21]	21/19 22/18	57~62 59~64	40/40	As_2_O_3_ (10 mg/d, qd) + TACE	TACE (ADM 20–40 mg/m^2^ + cis-platinum 20–40 mg/m^2^ + iodised oil, iv, 28 days)	Clinical effect, AFP, survival	E: NR C: NR

E, experimental group for As_2_O_3_ with TACE in treating PHC; C, control group for TACE in treating PHC; NR, not reported; MMC, mitomycin; ADM, adriamycin; Epi-ADM, Epirubicin Hydrochloride; HCPT, hydroxy camptothecin; 5-FU, 5-fluorouracil; CAP, carboplatin; THP, pirarubicin.

**Table 2:** The quality assessment facts of inclusion in literature.

Included studies	A	B	C	D	E	F	G
Qi et al., 2003 [13]	Unclear	Unclear	No	No	Unclear	Unclear	Unclear
Cui et al., 2006 [7]	Unclear	Unclear	No	No	Unclear	Unclear	Unclear
Zhuang et al., 2006 [10]	Yes	Unclear	No	No	Yes	Unclear	Unclear
Xie et al., 2007 [14]	Unclear	Unclear	No	No	Unclear	Unclear	Unclear
Zhou et al., 2007 [15]	Unclear	Unclear	No	No	Unclear	Unclear	Unclear
Wang, 2012 [5]	Unclear	Unclear	No	No	Unclear	Unclear	Unclear
Kui et al., 2010 [11]	Unclear	Unclear	No	No	Yes	Unclear	Unclear
Huang, 2011 [4]	Yes	Yes	No	Yes	Unclear	Unclear	Unclear
Zhang et al., 2011 [12]	Unclear	Unclear	No	No	Yes	Unclear	Unclear
Meng et al., 2012 [8]	Yes	Unclear	No	No	Unclear	Unclear	Unclear
Xing, 2012 [9]	Yes	Unclear	No	No	Unclear	Unclear	Unclear
Hu et al., 2014 [16]	Unclear	Unclear	No	No	Unclear	Unclear	Unclear
Qian, 2014 [17]	Unclear	Unclear	No	No	Unclear	Unclear	Unclear
Wan et al., 2014 [18]	Unclear	Unclear	No	No	Unclear	Unclear	Unclear
Xiang, 2014 [19]	Unclear	Unclear	No	No	Unclear	Unclear	Unclear
Meng et al., 2015 [20]	Unclear	Unclear	No	No	Unclear	Unclear	Unclear
Yang and Li, 2015 [21]	Unclear	Unclear	No	No	Unclear	Unclear	Unclear

Note: A: random sequence generation (selection bias); B: allocation concealment (selection bias); C: blinding of participants and personnel (performance bias); D: blinding of outcome assessment (detection bias); E: incomplete outcome data; F: selective reporting (reporting bias); G: other biases.
